# Supplementary material for: The miR-96 and RARγ signaling axis governs androgen signaling and prostate cancer progression
Source: Oncogene. 2018 Aug 17;38(3):421–44. doi: 10.1038/s41388-018-0450-6 (PMC6336686; doi:10.1038/s41388-018-0450-6)
Supplement: Supplementary file 1 — Supp Figs [file 41388_2018_450_MOESM1_ESM.pptx]

## Slide 1
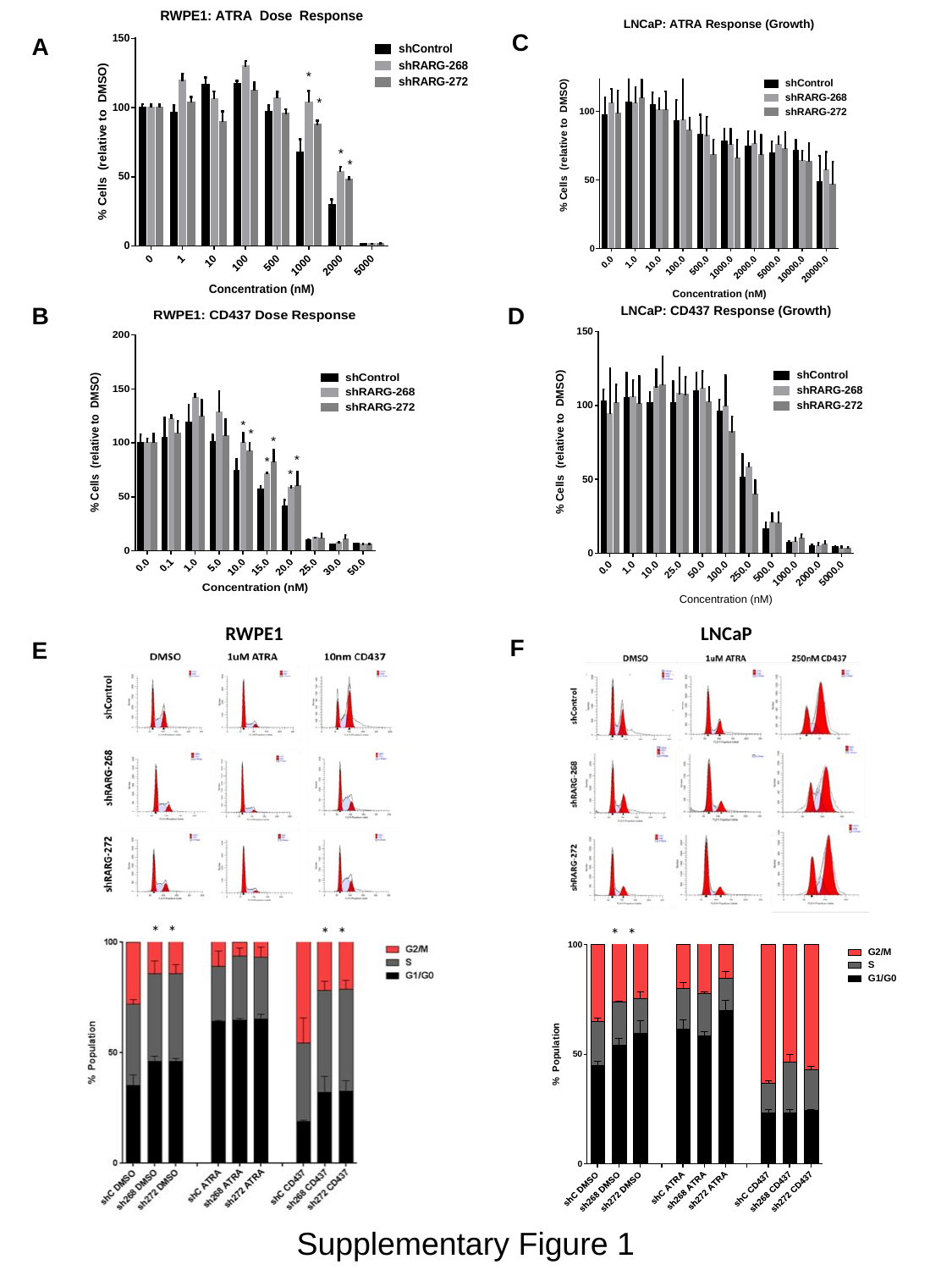

C
C
A
B
D
RWPE1
LNCaP
F
E
* *
* *
* *
Supplementary Figure 1

## Slide 2
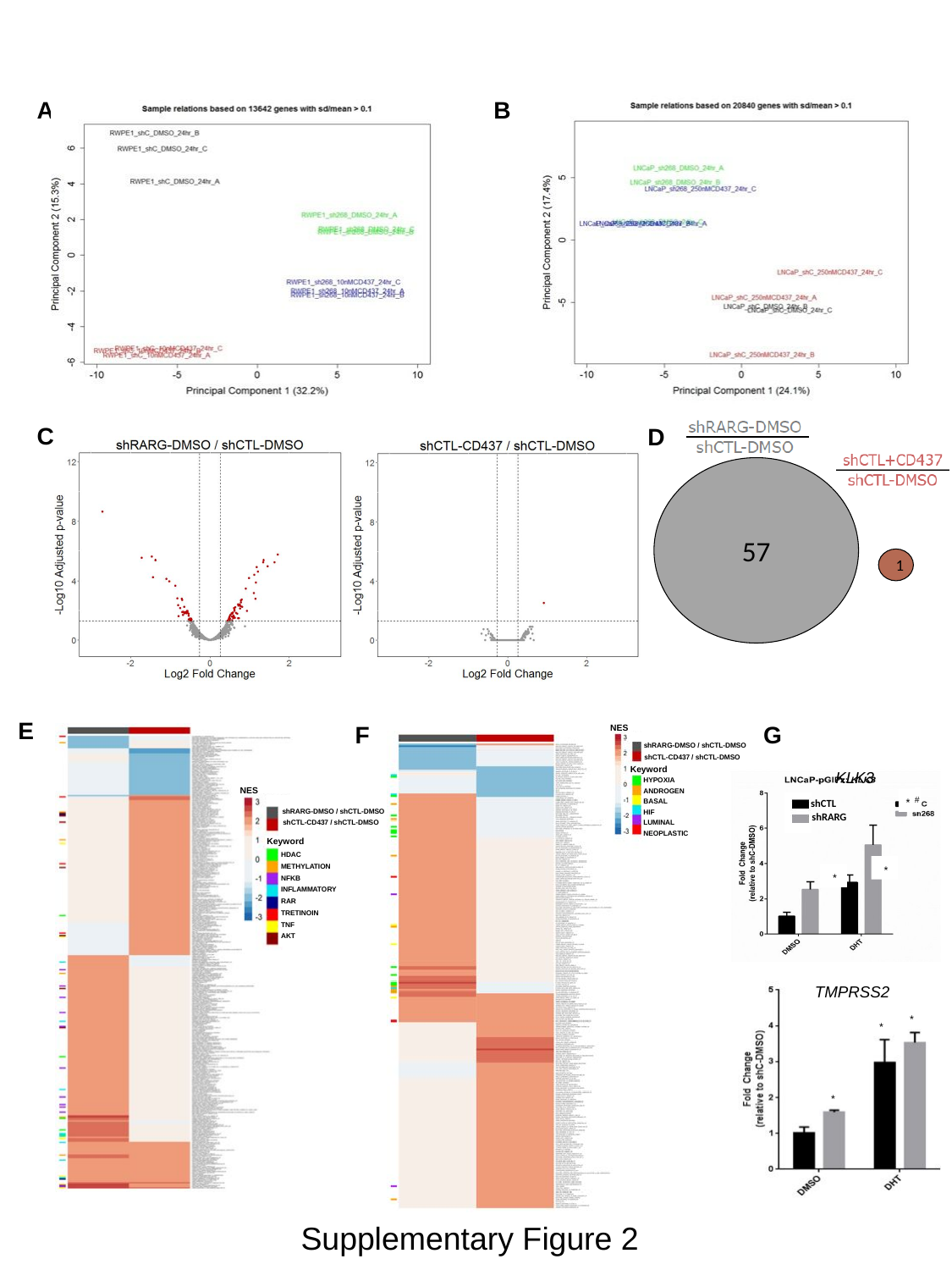

B
A
C
D
57
1
E
F
G
NES
shRARG-DMSO / shCTL-DMSO
shCTL-CD437 / shCTL-DMSO
Keyword
KLK3
*
*
*
#
TMPRSS2
*
*
*
shCTL
shRARG
HYPOXIA
ANDROGEN
BASAL
HIF
LUMINAL
NEOPLASTIC
NES
shRARG-DMSO / shCTL-DMSO
shCTL-CD437 / shCTL-DMSO
Keyword
HDAC
METHYLATION
NFKB
INFLAMMATORY
RAR
TRETINOIN
TNF
AKT
Supplementary Figure 2

## Slide 3
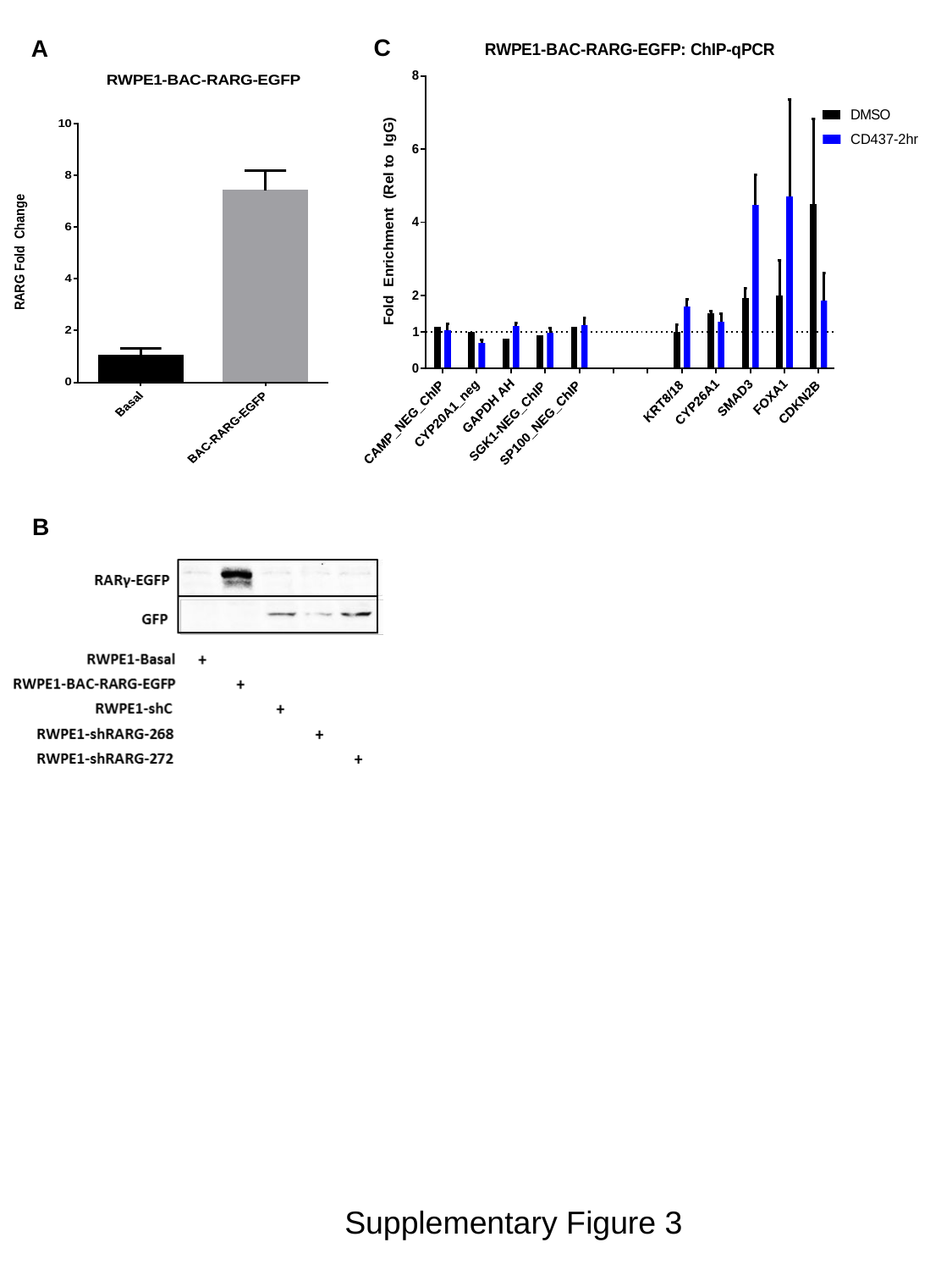

C
A
B
Supplementary Figure 3

## Slide 4
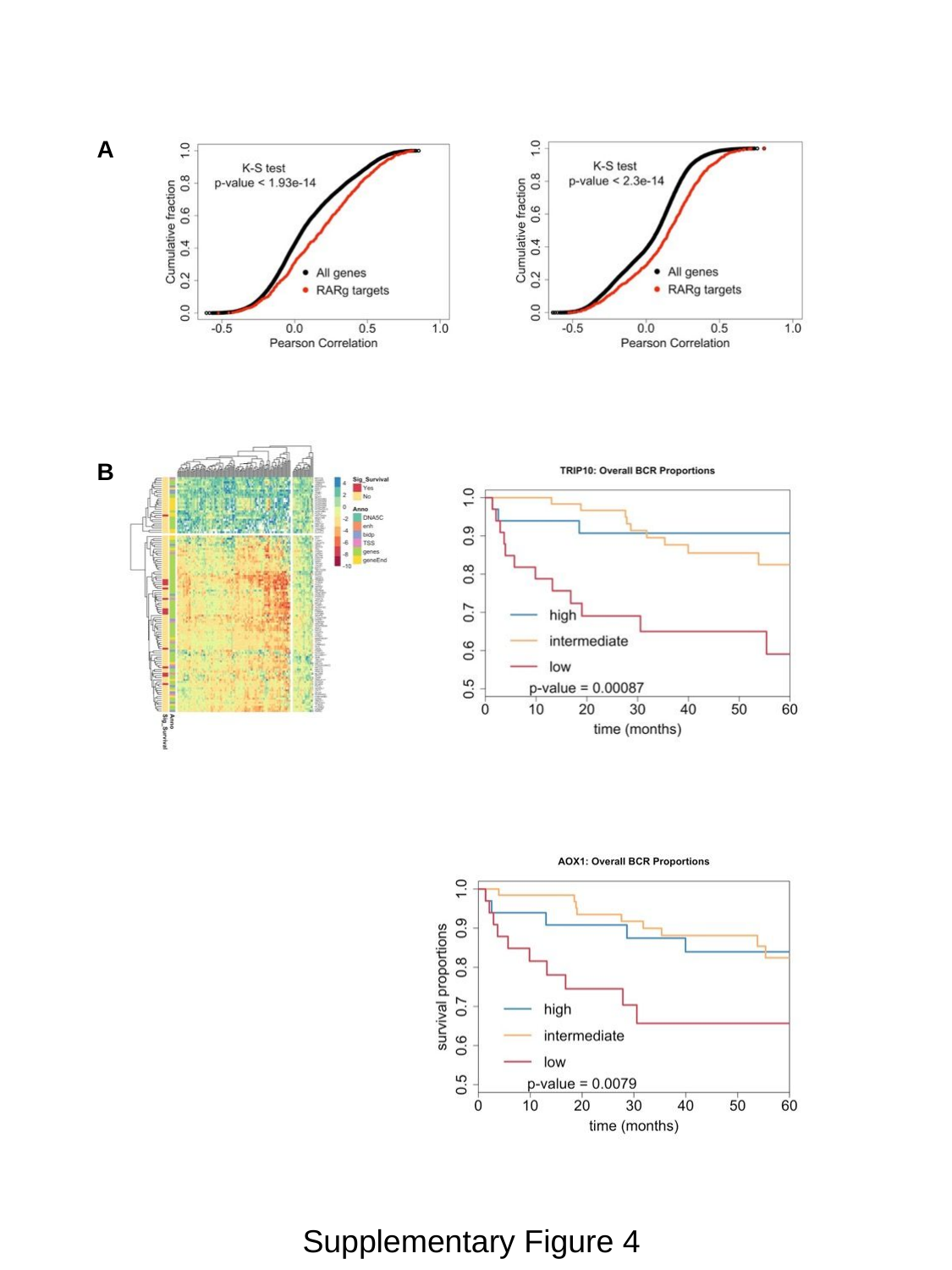

A
B
Supplementary Figure 4

## Slide 5
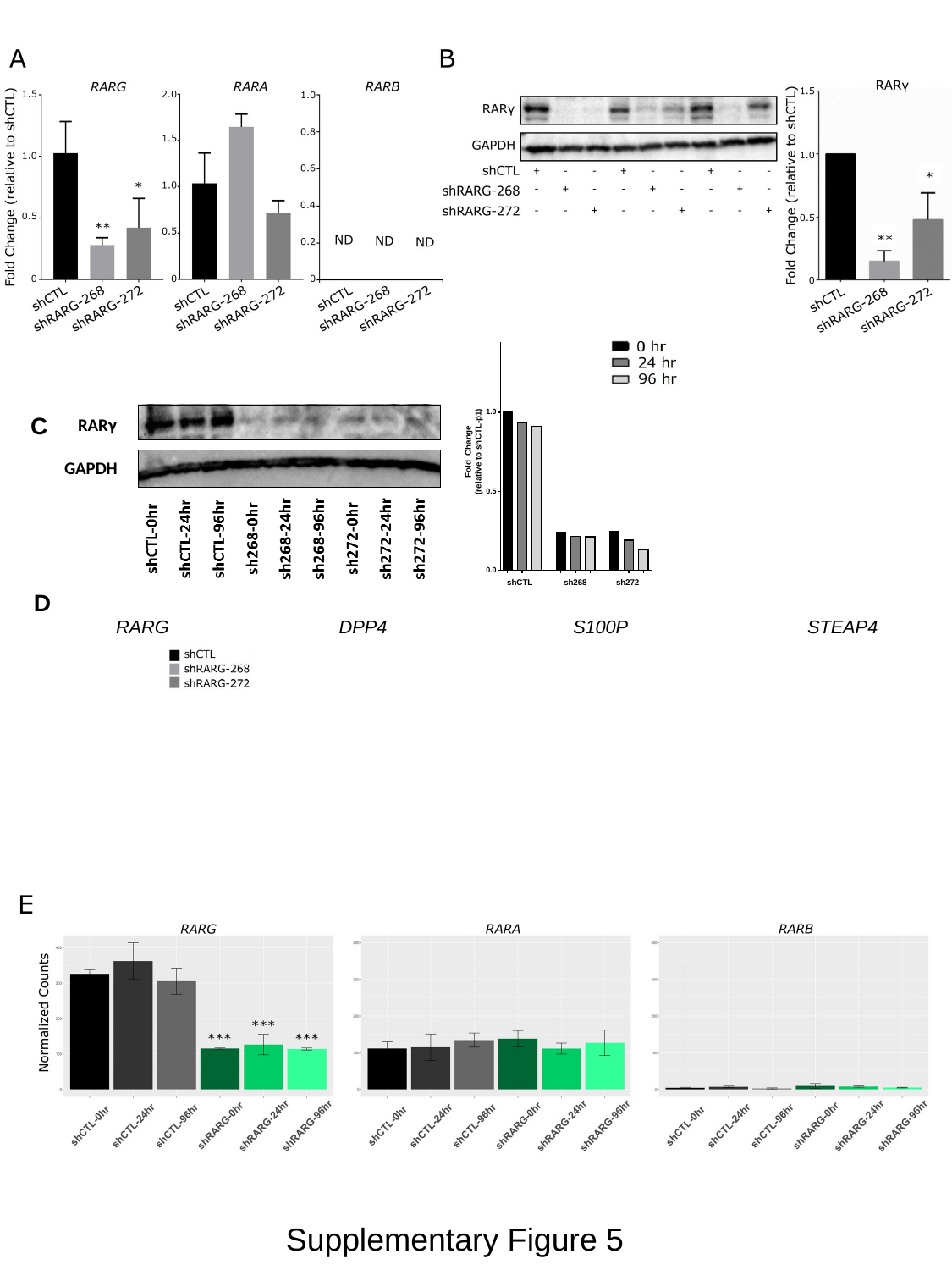

C
RARγ
GAPDH
D
RARG
DPP4
S100P
STEAP4
Supplementary Figure 5

## Slide 6
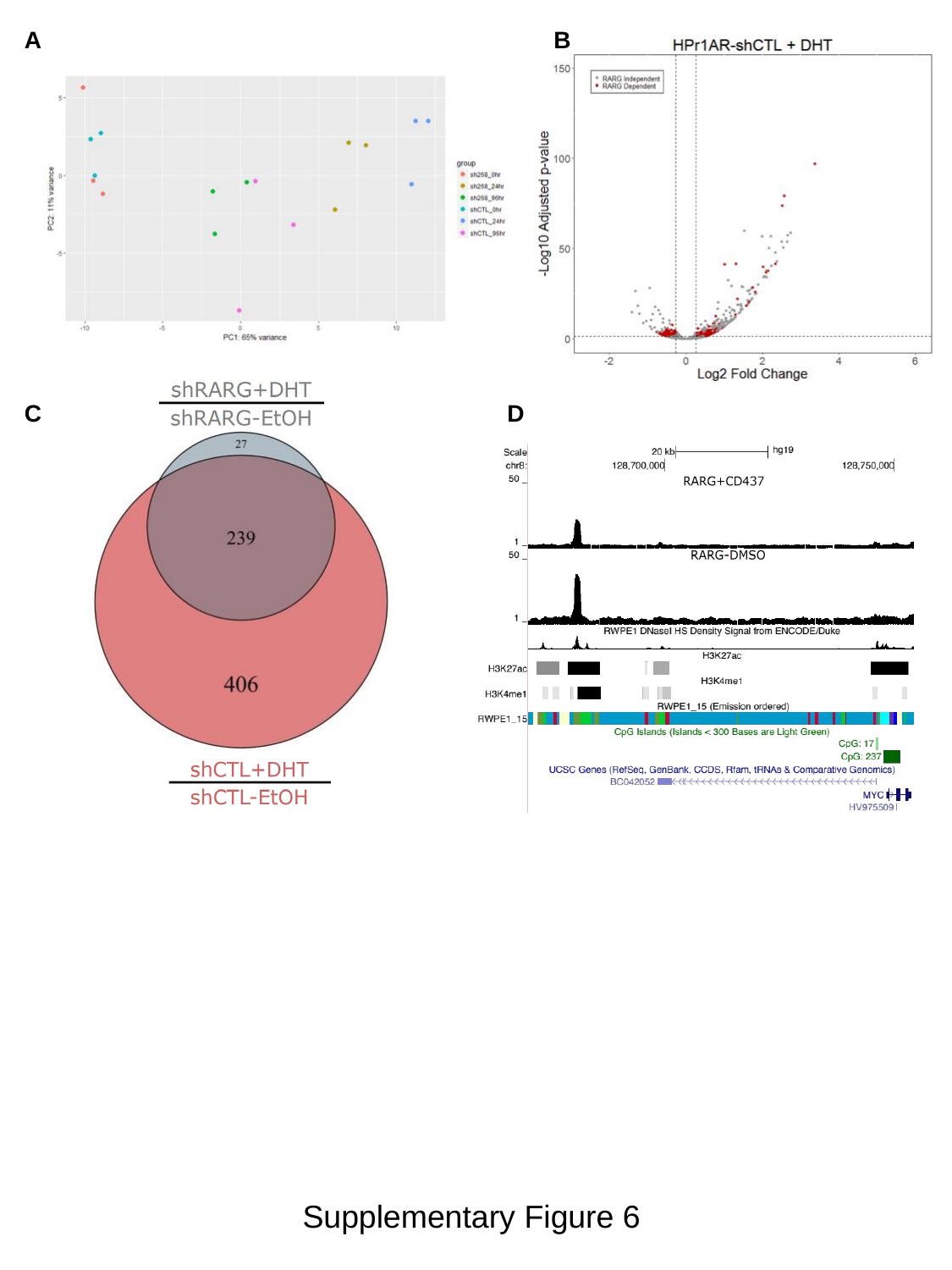

A
B
C
D
Supplementary Figure 6

## Slide 7
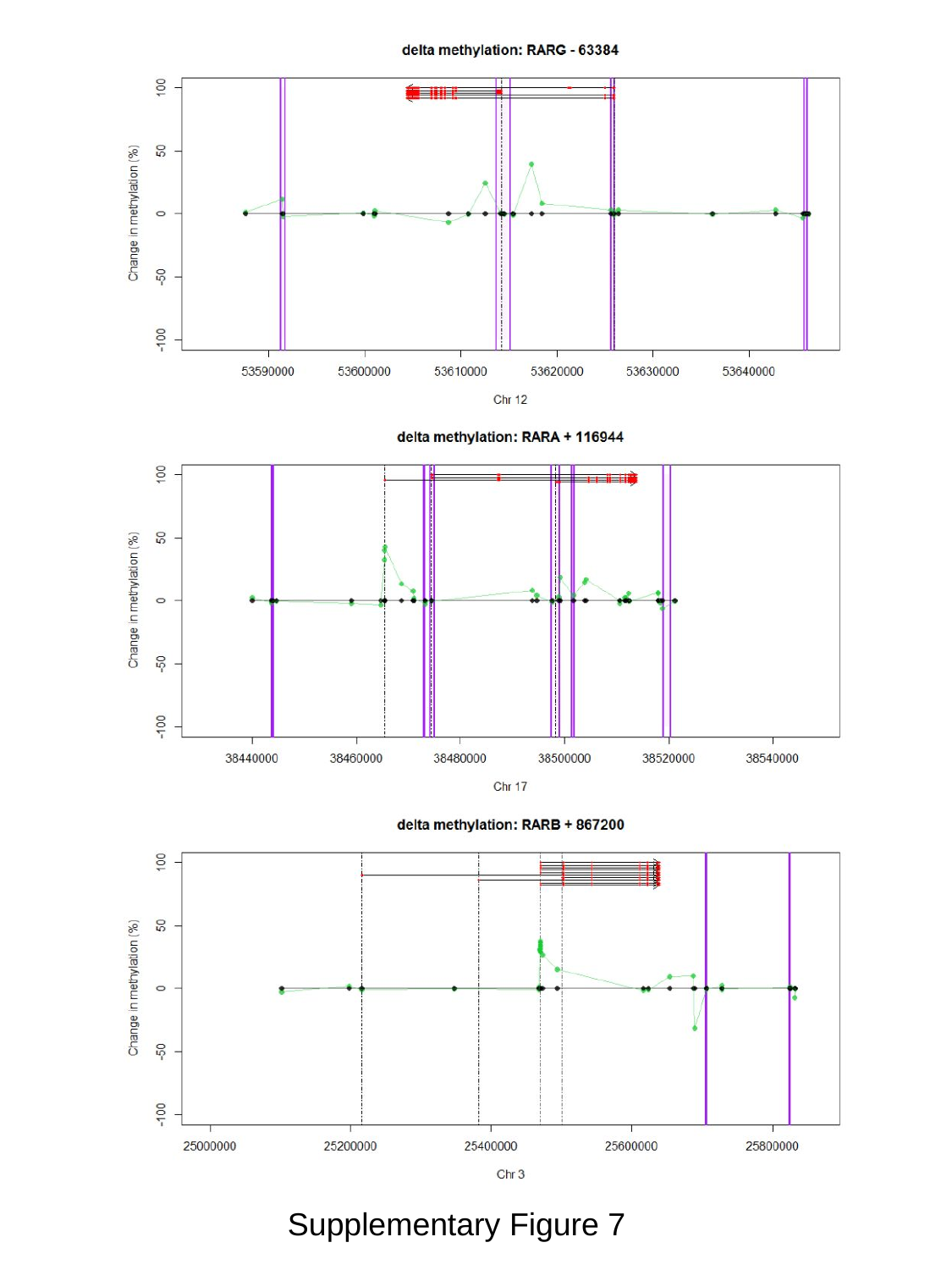

Supplementary Figure 7

## Slide 8
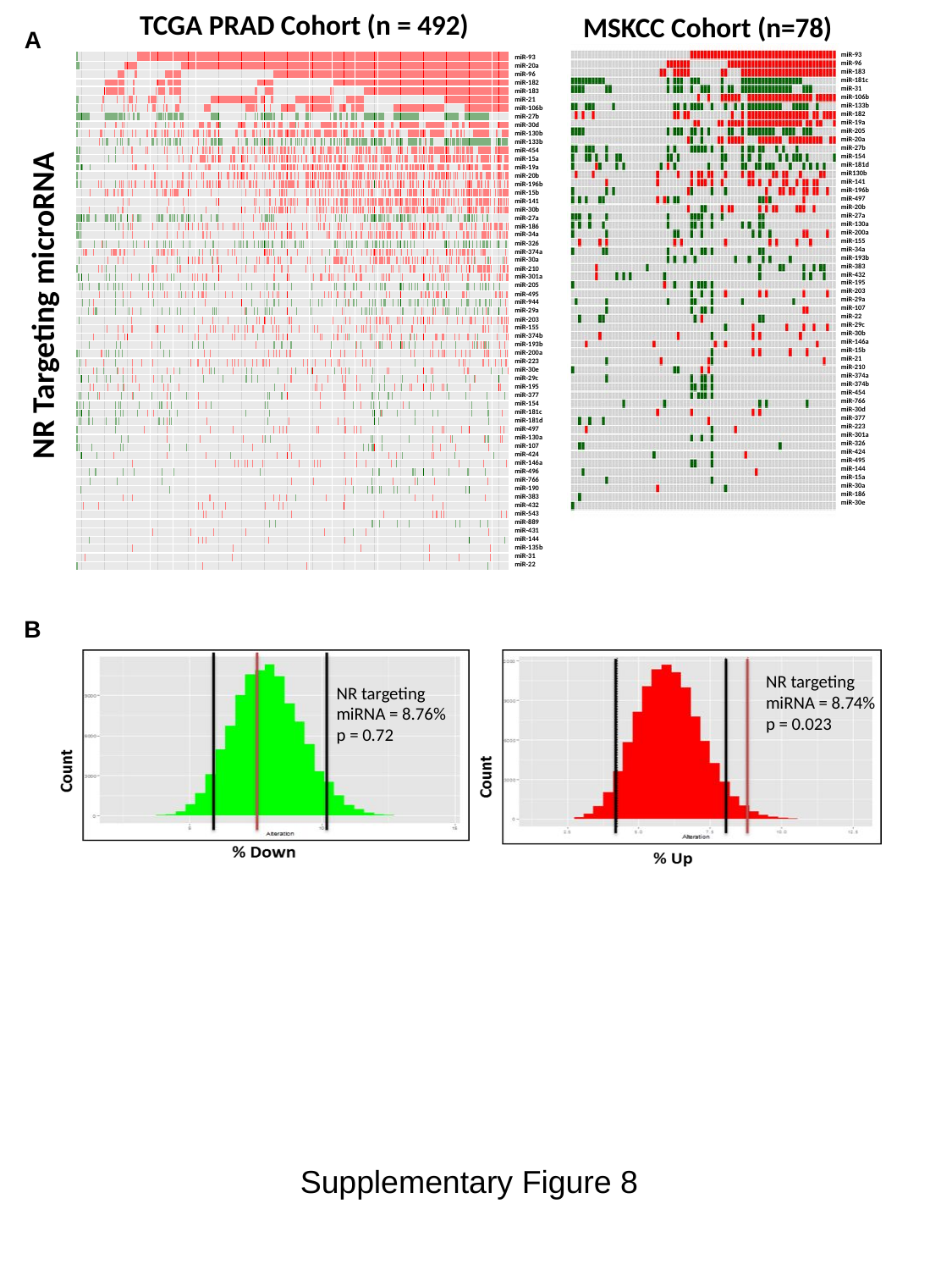

TCGA PRAD Cohort (n = 492)
MSKCC Cohort (n=78)
miR-93
miR-96
miR-183
miR-181c
miR-31
miR-106b
miR-133b
miR-182
miR-19a
miR-205
miR-20a
miR-27b
miR-154
miR-181d
miR130b
miR-141
miR-196b
miR-497
miR-20b
miR-27a
miR-130a
miR-200a
miR-155
miR-34a
miR-193b
miR-383
miR-432
miR-195
miR-203
miR-29a
miR-107
miR-22
miR-29c
miR-30b
miR-146a
miR-15b
miR-21
miR-210
miR-374a
miR-374b
miR-454
miR-766
miR-30d
miR-377
miR-223
miR-301a
miR-326
miR-424
miR-495
miR-144
miR-15a
miR-30a
miR-186
miR-30e
NR Targeting microRNA
A
miR-93
miR-20a
miR-96
miR-182
miR-183
miR-21
miR-106b
miR-27b
miR-30d
miR-130b
miR-133b
miR-454
miR-15a
miR-19a
miR-20b
miR-196b
miR-15b
miR-141
miR-30b
miR-27a
miR-186
miR-34a
miR-326
miR-374a
miR-30a
miR-210
miR-301a
miR-205
miR-495
miR-944
miR-29a
miR-203
miR-155
miR-374b
miR-193b
miR-200a
miR-223
miR-30e
miR-29c
miR-195
miR-377
miR-154
miR-181c
miR-181d
miR-497
miR-130a
miR-107
miR-424
miR-146a
miR-496
miR-766
miR-190
miR-383
miR-432
miR-543
miR-889
miR-431
miR-144
miR-135b
miR-31
miR-22
B
NR targeting
miRNA = 8.74%
p = 0.023
NR targeting
miRNA = 8.76%
p = 0.72
Supplementary Figure 8

## Slide 9
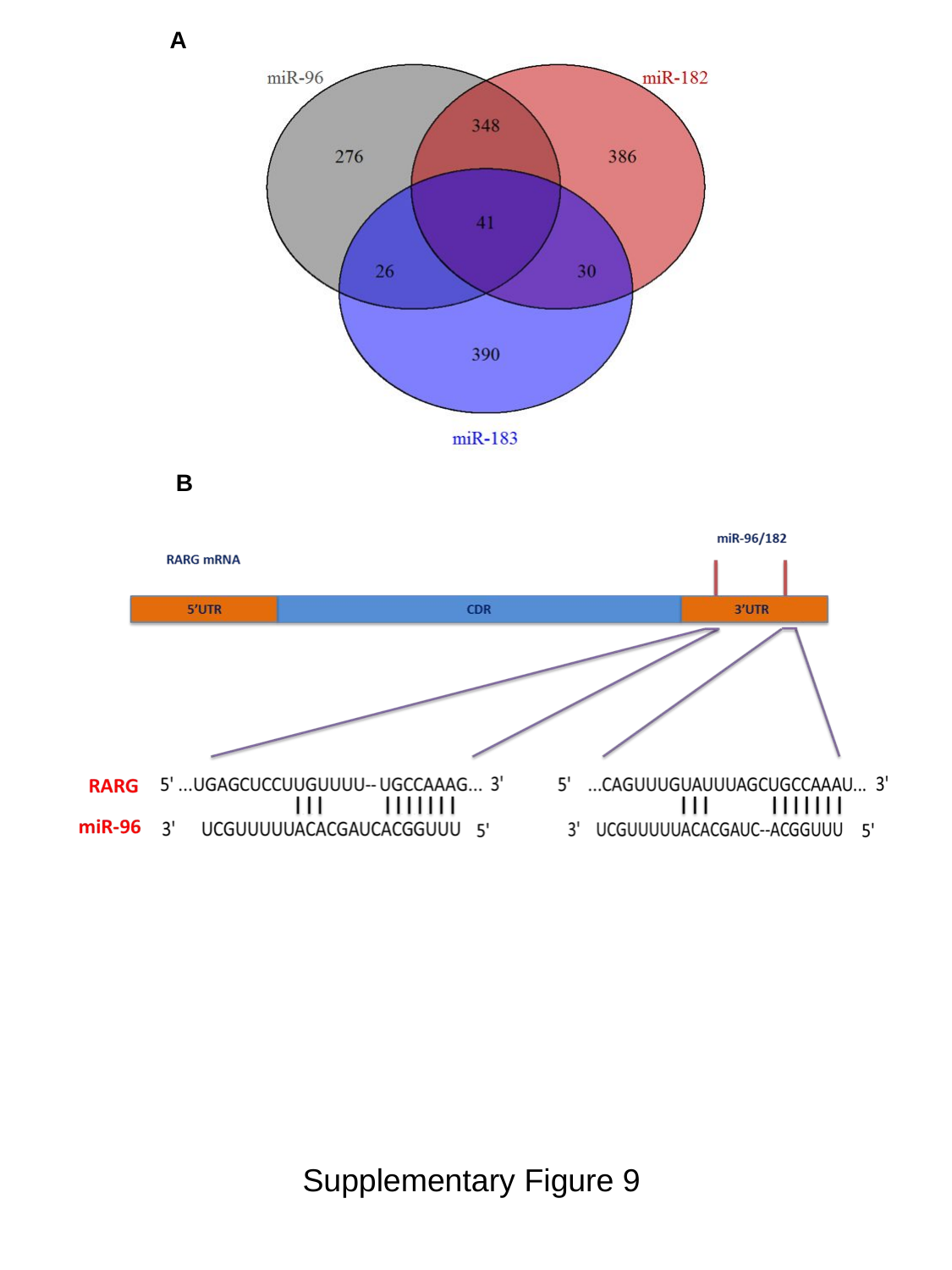

A
B
Supplementary Figure 9

## Slide 10
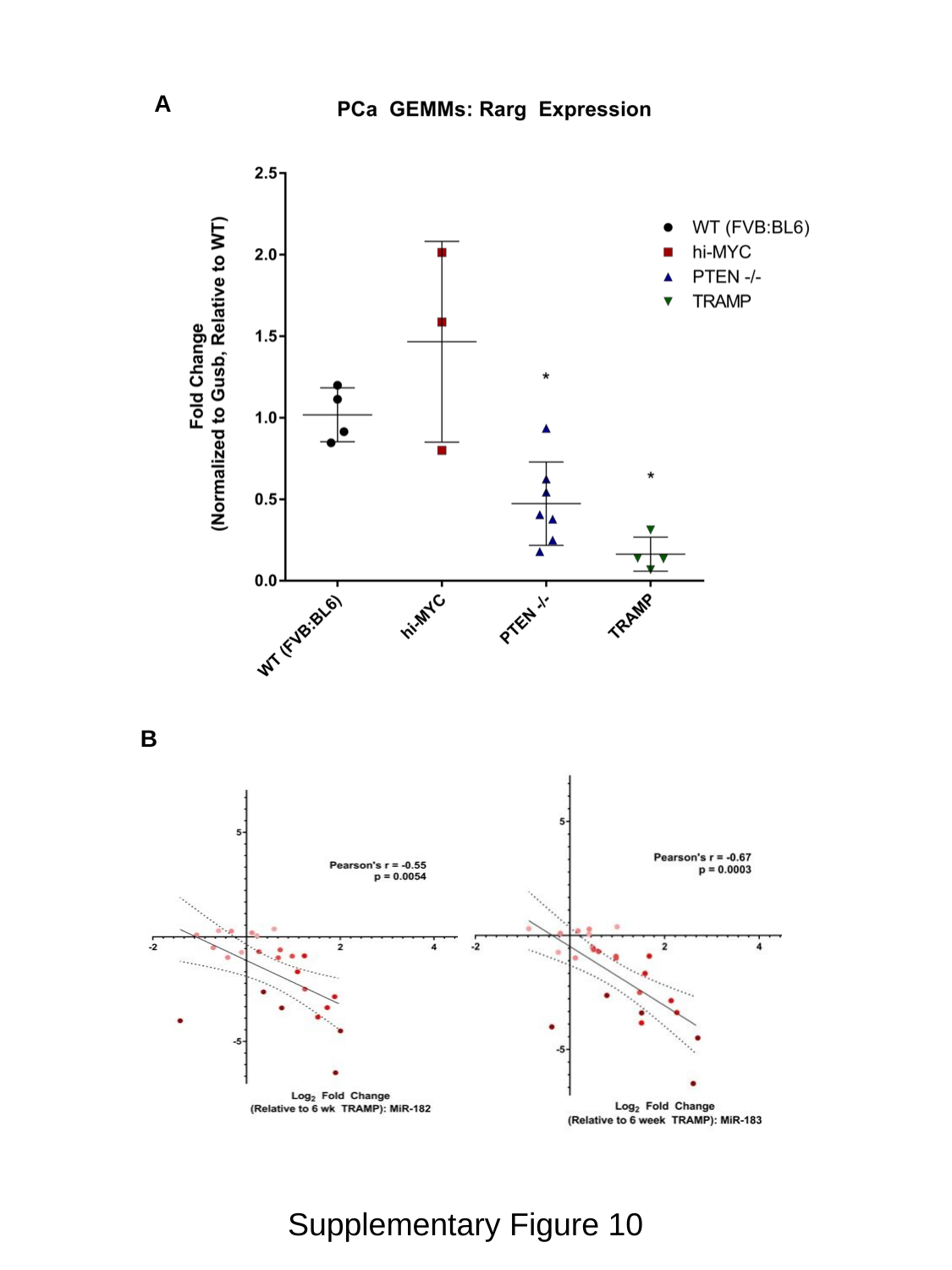

A
B
Supplementary Figure 10

## Slide 11
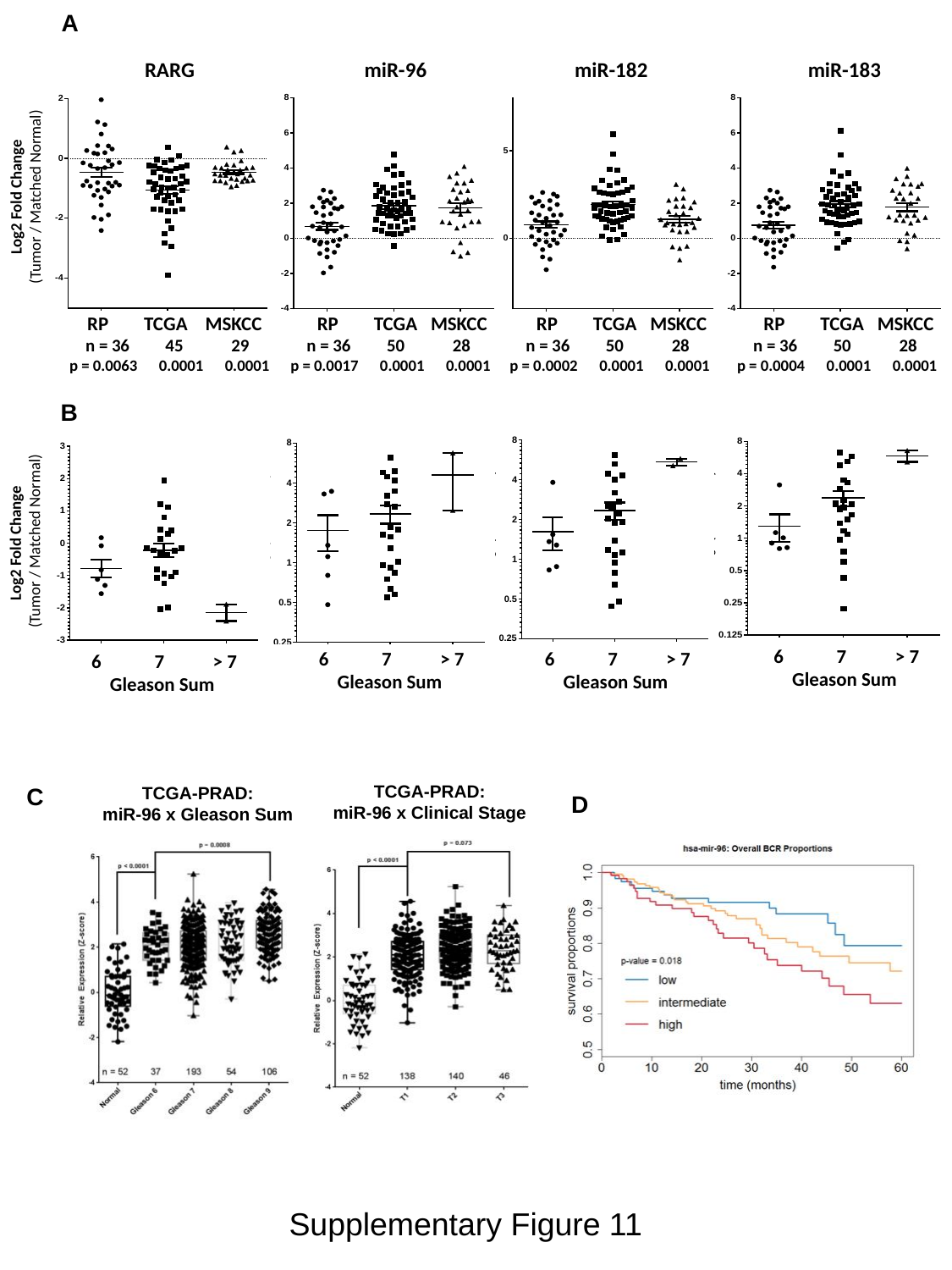

A
miR-182
miR-183
RARG
miR-96
Log2 Fold Change
(Tumor / Matched Normal)
 RP TCGA MSKCC
 n = 36 45 29
p = 0.0063 0.0001 0.0001
 RP TCGA MSKCC
 n = 36 50 28
p = 0.0017 0.0001 0.0001
 RP TCGA MSKCC
 n = 36 50 28
p = 0.0002 0.0001 0.0001
 RP TCGA MSKCC
 n = 36 50 28
p = 0.0004 0.0001 0.0001
B
Log2 Fold Change
(Tumor / Matched Normal)
 6 7 > 7
Gleason Sum
 6 7 > 7
Gleason Sum
 6 7 > 7
Gleason Sum
 6 7 > 7
Gleason Sum
TCGA-PRAD:
miR-96 x Clinical Stage
C
TCGA-PRAD:
miR-96 x Gleason Sum
D
Supplementary Figure 11

## Slide 12
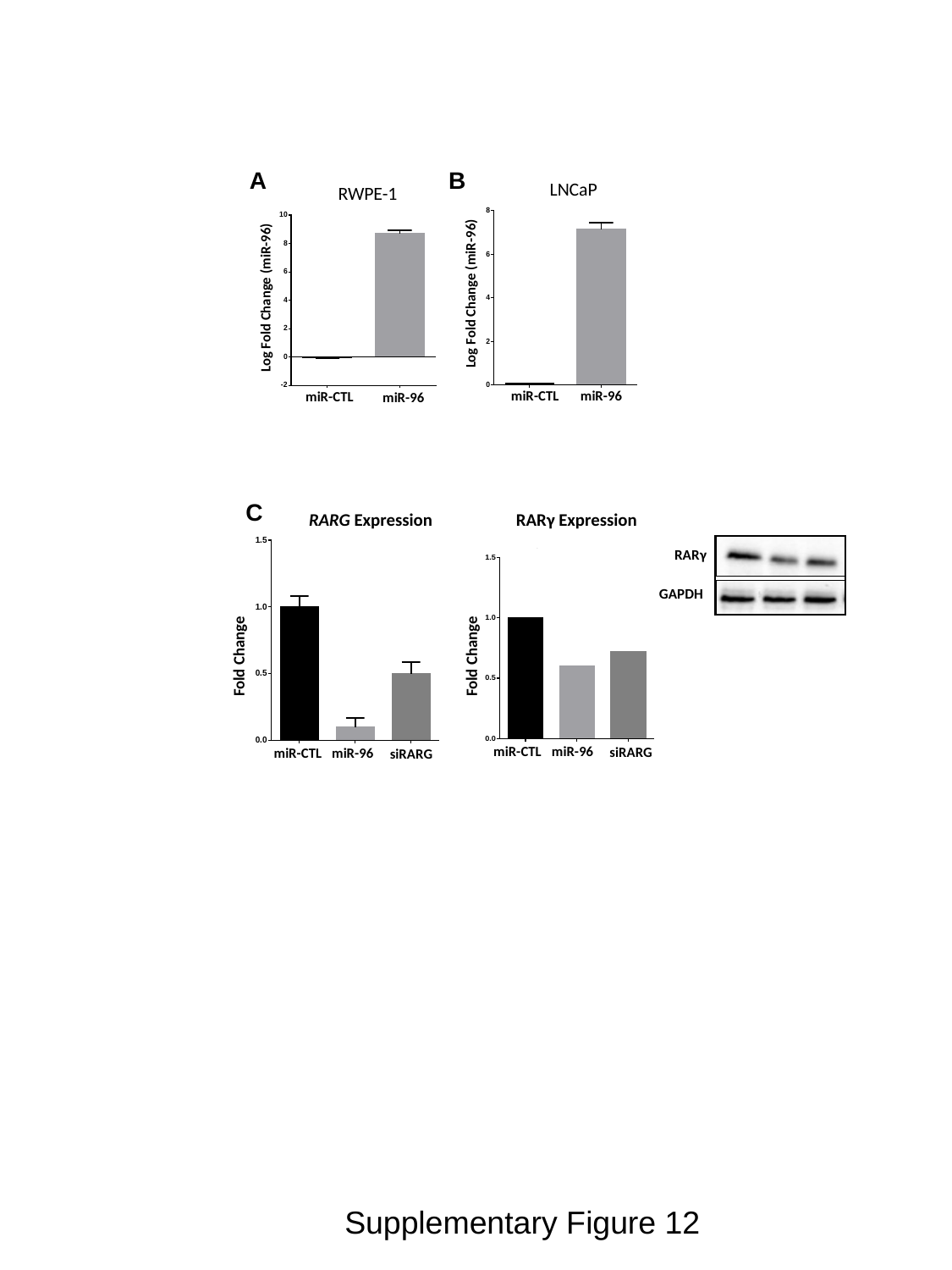

B
A
LNCaP
RWPE-1
Log Fold Change (miR-96)
Log Fold Change (miR-96)
miR-96
miR-CTL
miR-CTL
miR-96
C
RARG Expression
RARγ Expression
RARγ
GAPDH
Fold Change
Fold Change
miR-CTL
miR-96
siRARG
miR-CTL
miR-96
siRARG
Supplementary Figure 12

## Slide 13
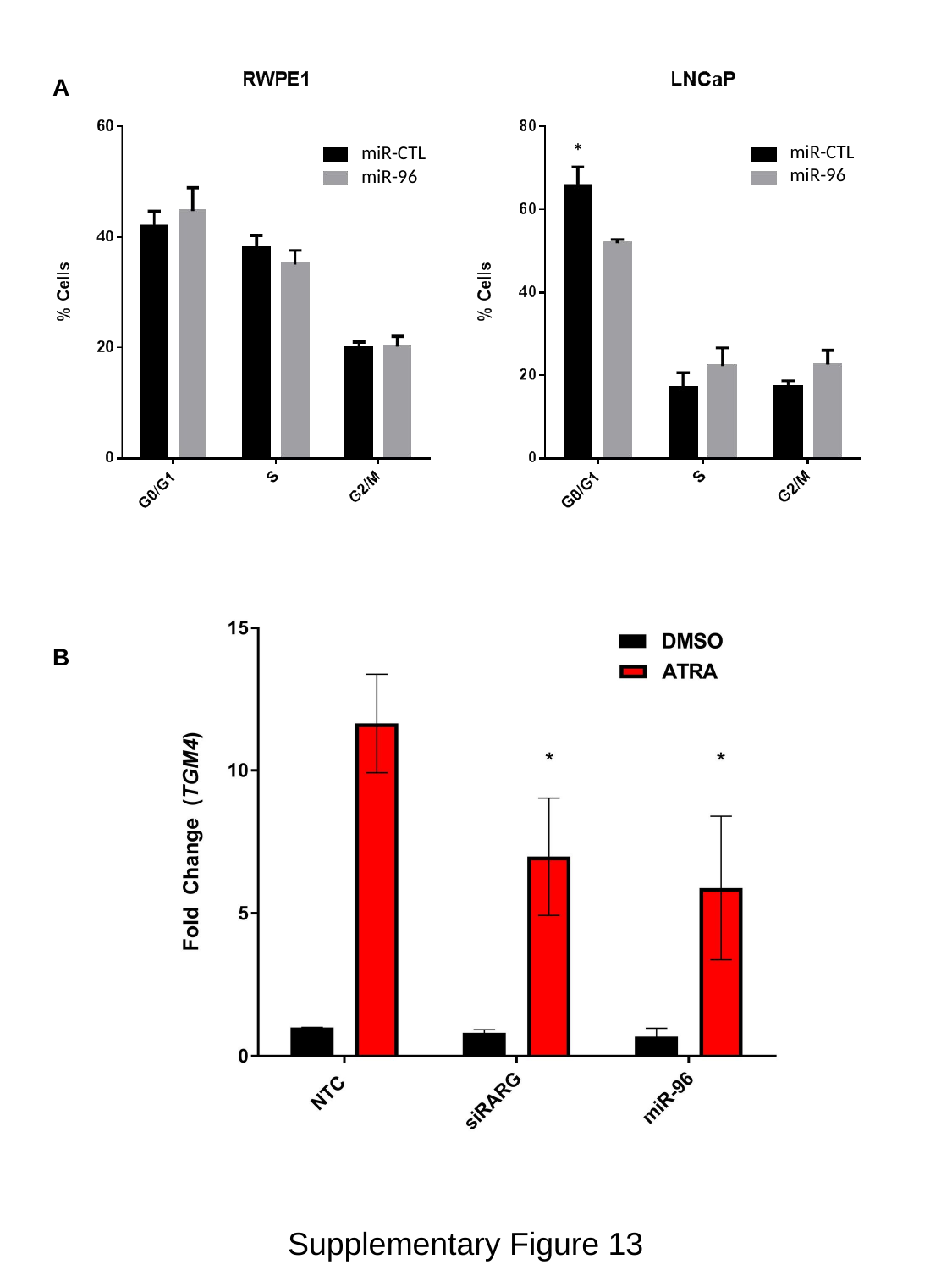

A
*
miR-CTL
miR-96
miR-CTL
miR-96
B
Supplementary Figure 13

## Slide 14
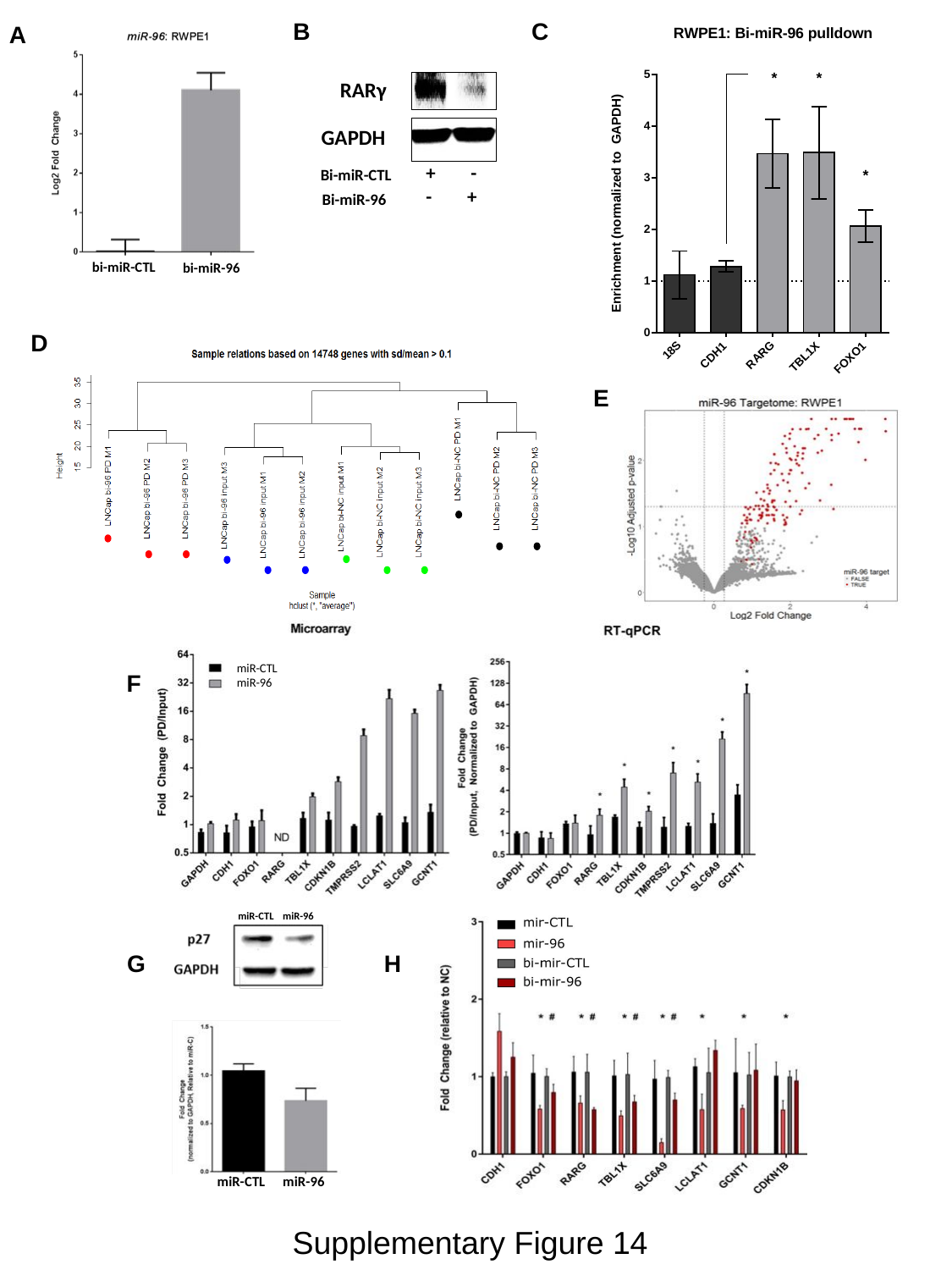

B
C
A
RARγ
GAPDH
 + -
 - +
Bi-miR-CTL
Bi-miR-96
bi-miR-CTL
bi-miR-96
D
E
miR-CTL
miR-96
F
miR-96
miR-CTL
G
H
miR-CTL
miR-96
Supplementary Figure 14

## Slide 15
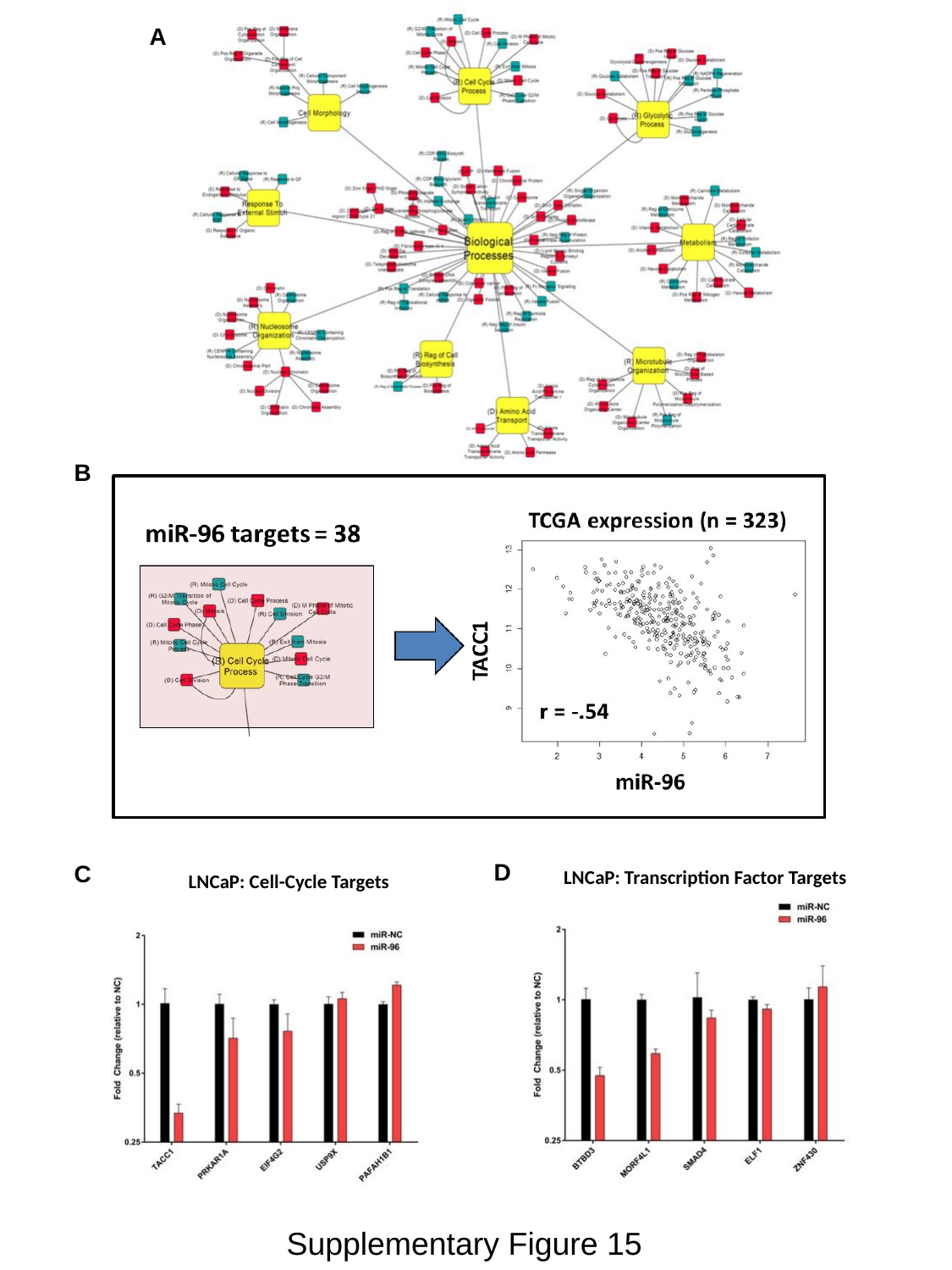

A
B
D
C
LNCaP: Transcription Factor Targets
LNCaP: Cell-Cycle Targets
Supplementary Figure 15

## Slide 16
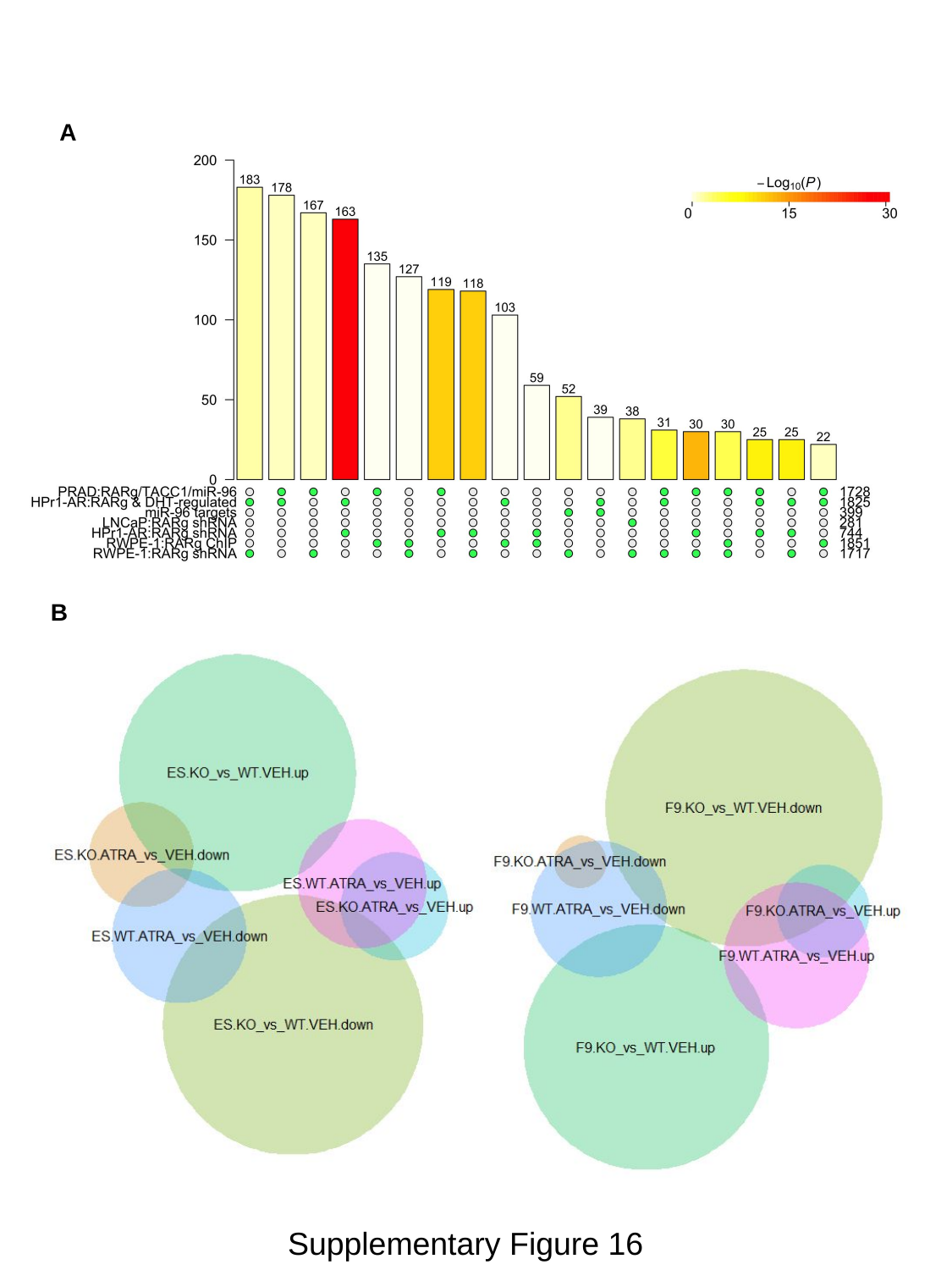

A
B
Supplementary Figure 16

## Slide 17
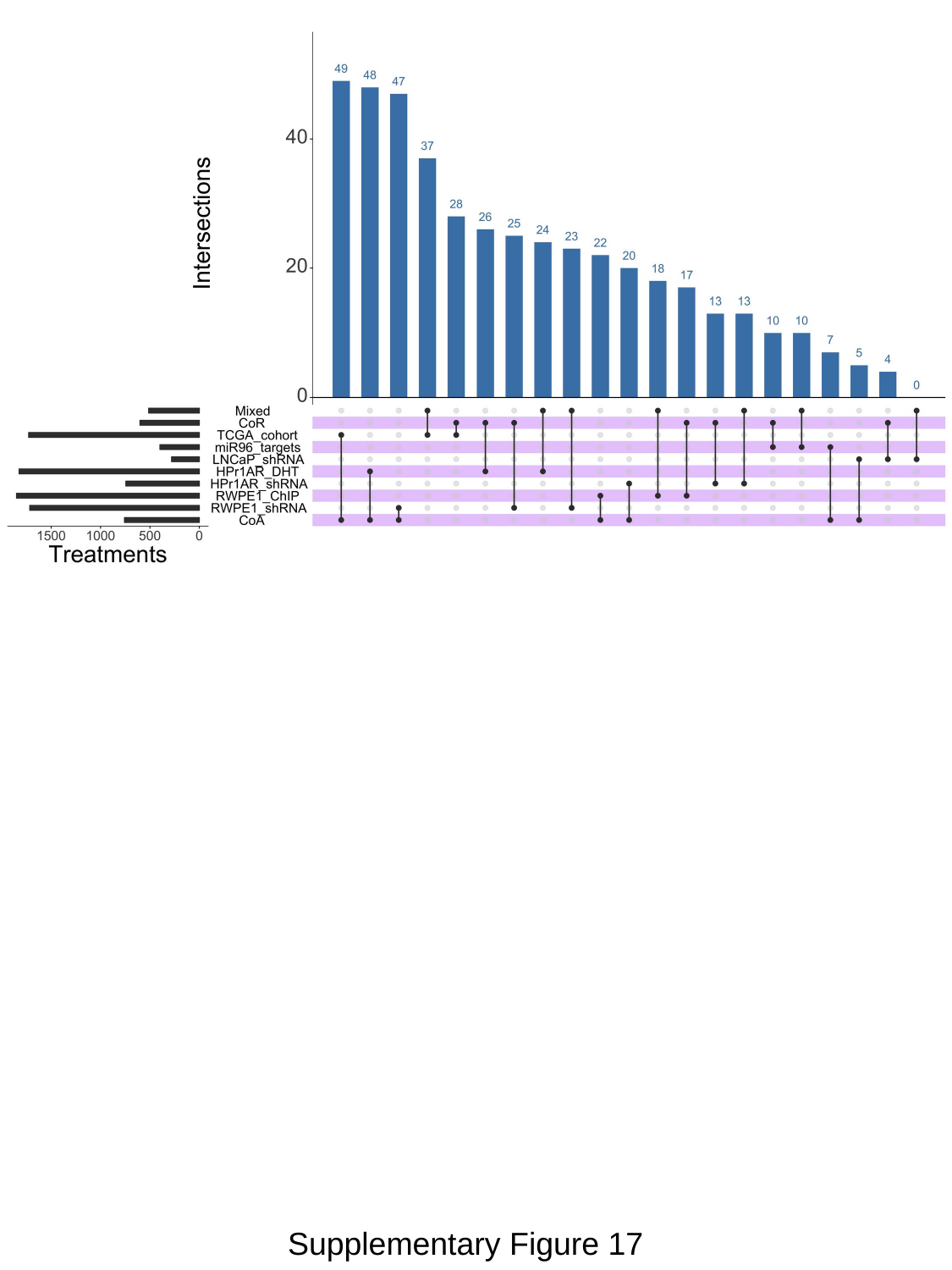

Supplementary Figure 17

## Slide 18
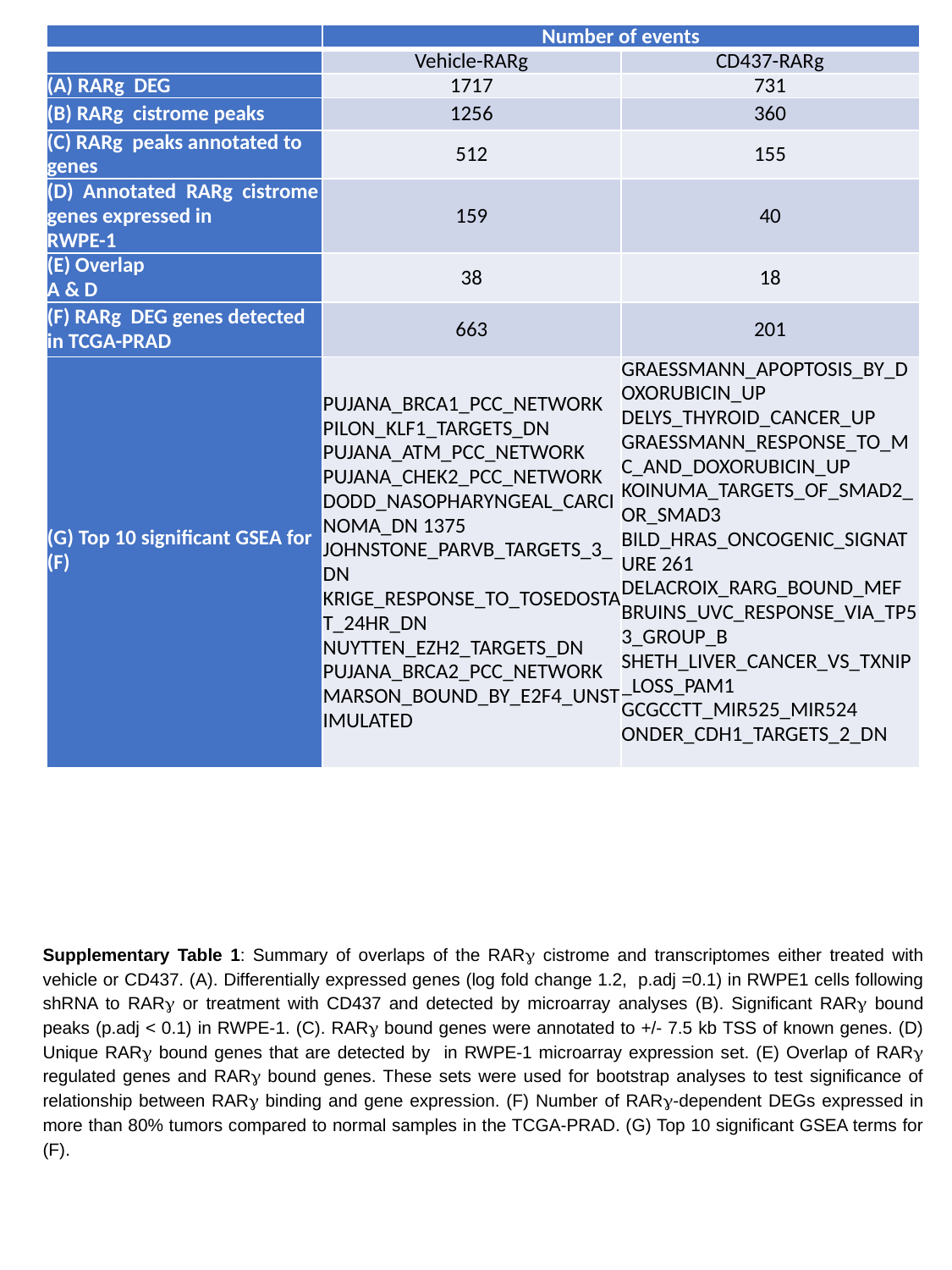

| | Number of events | |
| --- | --- | --- |
| | Vehicle-RARg | CD437-RARg |
| (A) RARg DEG | 1717 | 731 |
| (B) RARg cistrome peaks | 1256 | 360 |
| (C) RARg peaks annotated to genes | 512 | 155 |
| (D) Annotated RARg cistrome genes expressed in RWPE-1 | 159 | 40 |
| (E) Overlap A & D | 38 | 18 |
| (F) RARg DEG genes detected in TCGA-PRAD | 663 | 201 |
| (G) Top 10 significant GSEA for (F) | PUJANA\_BRCA1\_PCC\_NETWORK PILON\_KLF1\_TARGETS\_DN PUJANA\_ATM\_PCC\_NETWORK PUJANA\_CHEK2\_PCC\_NETWORK DODD\_NASOPHARYNGEAL\_CARCINOMA\_DN 1375 JOHNSTONE\_PARVB\_TARGETS\_3\_DN KRIGE\_RESPONSE\_TO\_TOSEDOSTAT\_24HR\_DN NUYTTEN\_EZH2\_TARGETS\_DN PUJANA\_BRCA2\_PCC\_NETWORK MARSON\_BOUND\_BY\_E2F4\_UNSTIMULATED | GRAESSMANN\_APOPTOSIS\_BY\_DOXORUBICIN\_UP DELYS\_THYROID\_CANCER\_UP GRAESSMANN\_RESPONSE\_TO\_MC\_AND\_DOXORUBICIN\_UP KOINUMA\_TARGETS\_OF\_SMAD2\_OR\_SMAD3 BILD\_HRAS\_ONCOGENIC\_SIGNATURE 261 DELACROIX\_RARG\_BOUND\_MEF BRUINS\_UVC\_RESPONSE\_VIA\_TP53\_GROUP\_B SHETH\_LIVER\_CANCER\_VS\_TXNIP\_LOSS\_PAM1 GCGCCTT\_MIR525\_MIR524 ONDER\_CDH1\_TARGETS\_2\_DN |
Supplementary Table 1: Summary of overlaps of the RARg cistrome and transcriptomes either treated with vehicle or CD437. (A). Differentially expressed genes (log fold change 1.2, p.adj =0.1) in RWPE1 cells following shRNA to RARg or treatment with CD437 and detected by microarray analyses (B). Significant RARg bound peaks (p.adj < 0.1) in RWPE-1. (C). RARg bound genes were annotated to +/- 7.5 kb TSS of known genes. (D) Unique RARg bound genes that are detected by in RWPE-1 microarray expression set. (E) Overlap of RARg regulated genes and RARg bound genes. These sets were used for bootstrap analyses to test significance of relationship between RARg binding and gene expression. (F) Number of RARg-dependent DEGs expressed in more than 80% tumors compared to normal samples in the TCGA-PRAD. (G) Top 10 significant GSEA terms for (F).
