# Supplementary material for: The miR-96 and RARγ signaling axis governs androgen signaling and prostate cancer progression
Source: Oncogene. 2018 Aug 17;38(3):421–44. doi: 10.1038/s41388-018-0450-6 (PMC6336686; doi:10.1038/s41388-018-0450-6)
Supplement: Supplementary file 2 — Supp Fig Legends [file 41388_2018_450_MOESM2_ESM.docx]

**Supplementary Figure 1:** Dose response curves were generated in RWPE1-shCTL and RWPE1-shRARG cell lines in responses to ATRA (**A**) and CD437 (**B**) for 96 hours. Significant differences in response relative to RWPE1-shCTL cells are indicated (p < 0.05). (**C,D**) Similar results shown for LNCaP-shCTL and LNCaP-shRARG cells treated for 144 hours. (**E**) Representative propidium iodide profiles of RWPE1-shCTL and RWPE1-shRARG cells treated with vehicle, 1 µM ATRA or 10nM CD437 for 96 hours. Cell cycle phase populations were determined by ModFit 3.1 software. Quantification of cell cycle profiles from triplicate experiments are shown below. Asterisks denote significant shifts in populations in shRARG cells relative to control cells in each treatment group (Student’s t-test, p < 0.05). (**F**): Similar results shown for LNCaP-shCTL and LNCaP-shRARG cells treated with vehicle, 1 µM ATRA or 250nM CD437 for 120 hours.

**Supplementary Figure 2:** (**A**) Principal component analysis depicting relationships amongst samples profiled by microarray in RWPE1 cells and (**B**) LNCaP cells. (**C**) Volcano plots depicting expression changes upon RARγ knockdown or in response to exogenous RARγ specific ligand (CD437) in LNCaP cells; dotted lines indicate DEG thresholds (p.adj < 0.05, fold change of +/- 1.2), and red dots represent RARγ dependent DEGs. (**D**) Venn diagram depicting number of determined DEGs associated with vehicle RARγ and ligand treated RARγ conditions. (**E**) (**F**) Heatmap depicting a summary of all significantly enriched pathways from GSEA (NES > 1.8, FDR q.val < 0.05) associated with vehicle RARγ and ligand treated RARγ conditions in RWPE-1 and LNCaP cells. Example enriched meta-groups amongst significant GSEA sets are shown as row annotations. (**G**) Expression of typical androgen regulated genes *KLK3* and *TMRPSS2* in RARγ knockdown LNCaP cells treated with 10nM DHT for 24 hours. Significant expression changes relative to LNCaP-shCTL DMSO treated cells is indicated (p < 0.05).

**Supplementary Figure 3: Validation of stable BAC-RARG-EGFP expressing cells.** (**A**) RARG transcript is detected at significantly elevated levels in RWPE1-BAC-RARG-EGFP cells relative to basal RWPE1 cells. (**B**) Detection of RARG-EGFP corresponding fusion protein in RWPE1-BAC-RARG-EGFP cells by use of GFP targeting antibody. RWPE1 cells containing pGIPz-shRNA vectors are used as control for detection of wild type GFP. (**C**) Candidate ChIP-qPCR in RWPE1-BAC-RARG-EGFP cells either treated with DMSO or 10nM CD437 for 2 hours at TSS regions of genes regulated by RARγ in RWPE-1 cells (CYP26A1, KRT8/18, CDKN2B, FOXA1, SMAD3) or negative control TSS regions at genes not regulated by RARγ.

**Supplementary Figure 4: MiR-96 expression in MSKCC and TCGA-PRAD cohorts. A.** Cumulative distribution plot comparing the correlations (Pearson’s r) between RARγ and all detectable genes from ChIP-Seq assay in cohort samples to the correlations of validated targets (red) across the same samples in TCGA-PRAD (left) and MSKCC (right). Significant difference between distributions is determined by Kolmogorov-Smirnov test. **B**. Filtering these gene sets as revealed which were most altered in the MSCKCC cohort. Several of these genes were independently associated with worse disease free survival

**Supplementary Figure 5:** (**A**) Validation of *RARG* mRNA and (**B**) RARγ protein knockdown in HPr1-AR-shCTL and HPr1AR-shRARG cells. (**C**) Protein levels of RARγ are unaffected by DHT treatment (10 nM, 24 and 96 hours). (**D**) Expression of *RARG* transcript and other candidate DHT regulated genes (*DPP4*, *STEAP4*, *S100P*) in HPr1AR cells. (**E**) Expression of *RARG*, *RARA*, *RARB* in RNA-Seq data from HPr1-AR cells.

**Supplementary Figure 6:** (**A**) Principal component analysis depicting relationships amongst samples profiled by RNA-seq in HPr1-AR cells. (**B**) Volcano plot representing the identification of RARγ dependent DHT induced (96hr) gene expression changes in HPr1-AR cells. (**C**) Corresponding Venn diagram. (**D**) Genomic locus including MYC TSS, depicting an upstream enhancer region with RARγ binding.

**Supplementary Figure 7:** Matching normal tissue relative DNA methylation changes in tumors from the TCGA-PRAD cohort. Each data point represents the average relative change in methylation for a given CpG site. The genomic loci for *RARG* (top), *RARA* (middle) and *RARB* (bottom) are shown. CpG islands are delineated in purple. The genomic distance (bp) and direction are indicated for each loci.

**Supplementary Figure 8:** (**A**): Putative NR targeting miRNA, identified via miRWalk, were compiled and normal tissue relative expression alterations determined in both TCGA-PRAD and MSKCC cohorts. (**B**): Bootstrapping analysis comparing expression alterations associated with putative NR targeting miRNA relative to all expressed miRNA in the TCGA-PRAD cohort.

**Supplementary Figure 9:** (**A**) Venn diagram comparing the number of predicted targets of members of the miR-96-182-183 cluster as determined by miRWalk. (**B**) Diagram depicting the *RARG* transcript and putative miR-96 target sites within the 3’UTR.

**Supplementary Figure 10:** (**A**) Prostate tissue was obtained from Hi-MYC, PTEN-/- and TRAMP mice and Rarg expression determined by RT-qPCR. Significant expression changes are indicated relative to wild type (WT) FVB:BL6 mice (p < 0.05). (**B**) Correlation analyses of *RARG* with miR-182 and miR-183 over the course of palpable tumor (PT) development in TRAMP. Time points are denoted by color. Pearson’s correlation coefficients are indicated for each comparison, as well as the significance of correlations.

**Supplementary Figure 11:** (**A**) Summary of expression changes observed within an independent cohort of 36 matched tumor/normal prostate samples (RPCCC) in relation to matching tumor/normal pairs from the TCGA-PRAD and MSKCC cohorts. Significance of expression changes in each case was determined via one sample t test. (**B**) Profile of expression changes in each tumor/matching normal pair relative to the Gleason sum for that patient in the RPCCC cohort. (**C**) miR-96 expression significantly increases with increasing Gleason sum and clinical stage within the TCGA-PRAD cohort. (**D**) Kaplan-Meier curves depicting the significantly reduced time to 5 year biochemical recurrence post radical prostatectomy in men with elevated miR-96 expression.

**Supplementary Figure 12:** (**A**) Validation of elevated miR-96 levels in RWPE1 cells and in (**B**) LNCaP cells after 48 hour transfection with miR-96 mimics. (**C**) *RARG* mRNA expression (left panel) and RARγ protein expression (right panels) in LNCaP cells after 48 hour transfections with miR-96 mimics or siRNA targeting RARG.

**Supplementary Figure 13:** Cell cycle distributions were assess in RWPE-1 (left) and LNCaP (right) cells 48 hours post-transfection with miR-96 or non-targeting control mimics. Significant differences in miR-96 mimic transfected cells relative to control cells is indicated (p < 0.05).

**Supplementary Figure 14:** (**A**): biotin labelled miR-96 is detectable at elevated levels relative to biotin labelled miRNA control by miRNA specific RT-qPCR after 24 hours of transfection in RWPE1 cells. (**B**): bi-miR-96 transfection results in loss of RARγ expression in RWPE1 cells. (**C**) Enrichment of known (*RARG*, *FOXO1*) and highly predicted (*TBL1X*) candidate targets after bi-miR-96 pulldown followed by RT-qPCR in RWPE1 cells. (**D**) Unsupervised hierarchical clustering of samples profiled for miR-96 target identification study. (**E**) Volcano plot depicting the enrichment of all genes in bi-miR-96 pulldown over input in RWPE-1 cells. Horizontal lines indicate an adjusted p-value = 0.05, and vertical lines indicate fold change of +/- 1.2, shown for reference. Genes marked in red (n = 111) were considered experimentally determined miR-96 direct targets, as they were significantly enriched in bi-miR-96 pulldown but not in bi-miR-NC pulldown. (**F**) A panel of genes was selected to assess the technical validity of target enrichment determined by microarray in LNCaP cells. Enrichment determined by microarray (left) is directly compared to enrichment determined by RT-qPCR (right). Statistically significant enrichment is indicated (p < 0.05). (**G**) Western blot showing p27 (*CDKN1B*) knockdown in LNCaP cells transfected with miR-96 for 48 hr. Representative blot shown (upper) with quantitation of triplicate blots (lower). (**H**) Either bi-miR-96 or non-biotinylated miR-96 mimics, and respective control mimics, were transfected in RWPE-1 cells for 48 hr and target gene expression examined. *CDH1* was assessed as a negative control, and specific targets were chosen either as they were either previously validated (*FOXO1*, *RARG*) were significantly enriched in bi-miR-chip assay. Significance is noted (p < 0.05), for comparisons of target transcripts in biotinylated-miR-96 (*#*) and non-biotinylated miR-96 (*) relative to respective controls.

**Supplementary Figure 15:** (**A**) Significant GO terms (adjusted p < 0.05) identified by cross-referencing miR-96 targets with curated gene sets associated with biological processes using DAVID Bioinformatics Resource 6.7 (blue) and topGO (red). Gene sets with similar annotations were grouped together and visualized using Cytoscape 3.2.0. Annotations not fitting into hierarchical groupings are placed under ‘Biological Processes’. (**B**) Diagram depicting strategy for revealing clinically meaningful miR-96-target relationships from GO term gene sets. As an example, a total of 38 miR-96 targets were found within enriched cell cycle related GO-terms. Correlations between these 38 genes and miR-96 were calculated across TCGA-PRAD cohort samples, for instance as seen for TACC1. The top 5 most significant, negative correlations were profiled for miR-96 regulation by RT-qPCR. This approach was applied to genes associated with cell cycle as described (**C**), and also for genes with functional annotations associated with transcription factors (**D**). Significance is noted (p < 0.05)

**Supplementary Figure 16: A.** The RARγ and miR-96 data sets generated in the current study were overlapped with each other using SuperExactTest^2^. **B.** Euler diagrams of publicaly available (GSE31280) transcriptomes in ES (top) or F9 (bottom) cells with RARγ -/- and treated with ATRA. Data were processed with limma and the circles represent the size and overlaps of the indicated transcriptomes.

**Supplementary Figure 17:** The RARγ and miR-96 data sets generated in the current study were overlapped with a comprehensive list of human transcription co-factors generated by mining FANTOM, Uniprot and the HGNO data bases as well as Gene Ontology terms related to transcriptional control. This generated a list of transcription factor co-repressors (n=601), co-activators (n=758) and mixed function co-regulators (n = 513). UpSetR^1^ was used to visualized the overlaps between these groups of co-factors with the conditional gene sets identified in the current study.

1 Conway JR, Lex A, Gehlenborg N. UpSetR: an R package for the visualization of intersecting sets and their properties. Bioinformatics 2017; 33: 2938-2940.

2 Wang M, Zhao Y, Zhang B. Efficient Test and Visualization of Multi-Set Intersections. Sci Rep 2015; 5: 16923.
